# Transvesicoscopic Bipolar Sealing of Vesicovaginal Fistula

**DOI:** 10.1089/cren.2018.0013

**Published:** 2018-06-01

**Authors:** Ali Akkoç, Cemil Aydın, Murat Uçar, Aykut Buğra Şentürk, Murat Topçuoğlu, Ahmet Metin

**Affiliations:** ^1^ Department of Urology, Faculty of Medicine, Alanya Alaaddin Keykubat University, Alanya, Turkey.; ^2^ Department of Urology, Faculty of Medicine, Hitit University, Corum, Turkey.; ^3^ Department of Urology, Faculty of Medicine, Abant Izzetbaysal University, Bolu, Turkey.

**Keywords:** sealing, vesicovaginal fistula, bipolar, vesicoscopic

## Abstract

***Introduction:*** Vesicovaginal fistula (VVF) is a problem that physically and psychologically debilitates the patient. Options for treatment of VVF include transabdominal, transvaginal, transvesical, laparoscopic, and robotic repair or minimally invasive methods such as fulguration. We describe a novel minimally invasive technique: transvesicoscopic bipolar sealing of the vesicovaginal fistula (TBSF).

***Case Presentation:*** We carried out the transvesicoscopic sealing of VVF with 5 mm of diameter on a 46-year-old woman, who had a failed conservative treatment with a Foley catheter placement. The patient was informed about the modified surgical procedure before operation. The fistula tract was sealed by using an electrothermal bipolar vessel sealer through a 5-mm transvesical ports. The patient was discharged on the first postoperative day and was on anticholinergic medications after the operation. The patient remained dry after the removal of the catheter at the third postoperative week.

***Conclusion:*** In select cases of VVF, TBSF may be effectively used for closure of the fistula tract.

## Introduction and Background

Vesicovaginal fistula (VVF) is a social and surgical problem for the female population. Owing to continuous urinary incontinence and smell of urine, these women are exposed to social ostracizing. The incidence of VVF varies between 0.3% and 2%. In developed countries, it is most commonly caused by gynecologic operations, particularly abdominal hysterectomy, meanwhile, in developing countries, inadequate obstetric care is the leading cause of VVF.^1^

VVF may be treated conservatively by bladder drainage in some cases, particularly in cases of small fistulas. When conservative treatment fails, surgical repair remains the only option. There is no best approach for patients with VVF. However, surgeons performing laparoscopic approach claim several advantages of laparoscopic repair such as shorter hospitalization, quicker recovery with earlier return to work, and better cosmetic results than the open surgical approach.

In this study, we present a novel technique by using an electrothermal bipolar vessel sealing system (EBVS) for repairing VVF.

## Case Presentation

The patient, a 46-year-old female, presented to our department with complaints of continuous urinary incontinence. Her medical history included laparoscopic hysterectomy because of uterine myoma 2 months before her visit. Complete blood count, routine biochemistry parameters, urinalysis, urine culture, and urinary system ultrasonography results were all normal. We identified a 5-mm wide fistula between the bladder trigone and the upper part of the vaginal vault with flexible cystoscopy at the outpatient clinic. The patient was catheterized for 3 weeks. The patient who had catheterization failure underwent transvesicoscopic bipolar sealing of the vesicovaginal fistula (TBSF) after 3 months from her primary gynecologic surgery. The patient signed an informed consent form after she was advised on the use of the novel modified surgical procedure.

### Surgical technique

**FIG. 1.**
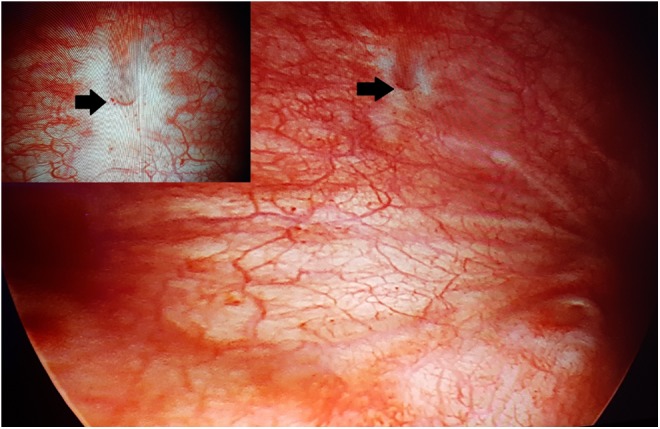
Cystoscopic appearance of small vesicovaginal fistula (*arrows*).

**FIG. 2.**
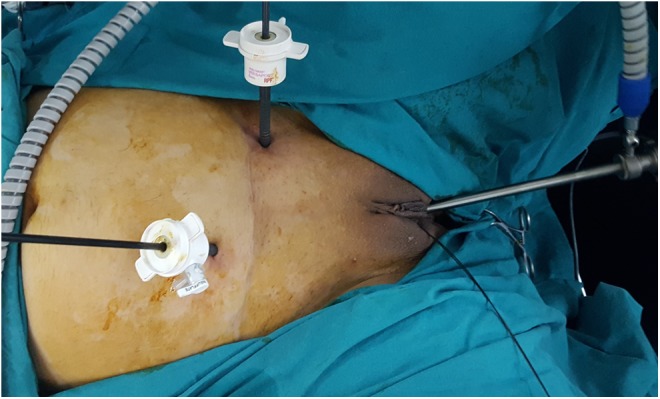
Port placement for transvesicoscopic sealing of fistula tract.

**FIG. 3.**
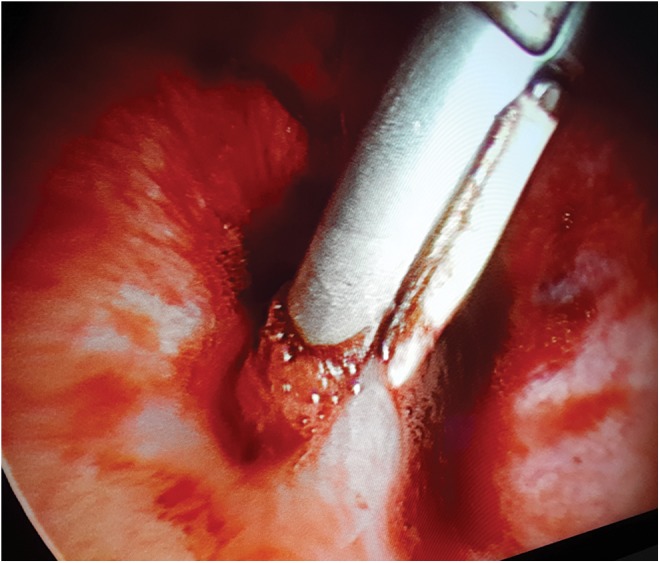
Sealing the fistula tract with the bipolar sealer.

The operation was conducted with the patient in lithotomy position under spinal anesthesia. A prophylactic antibiotic (second-generation cephalosporin) was given half an hour before the induction. The VVF was identified with a cystoscope using insufflation of gas and it was verified by a guidewire (Fig. 1). The vagina was packed with vaseline-soaked gauze to prevent leakage during bladder filling and escape of CO_2_ during vesicoscopy. A 5-mm laparoscopic port was placed into the bladder under cystoscopic guidance in the lateral of the midline, halfway between the umbilicus and symphysis pubis. The second 5-mm port was placed into the bladder laterally at the midline and inferior to the first port (Fig. 2). A cystoscope was used as a transurethral camera for vesicoscopy. Bladder mucosa and muscular layer were gripped with a forceps and raised up for a multilayer closure. The fistula tract was grasped and sealed by an EBVS (LigaSure™ 5 mm blunt tip 37 cm sealer; Medtronic, Inc., Dublin, Ireland) (Fig. 3). An 18F Ryle's tube was kept in the bladder as a cystostomy through one of the existent ports. The bladder was also catheterized.

**FIG. 4.**
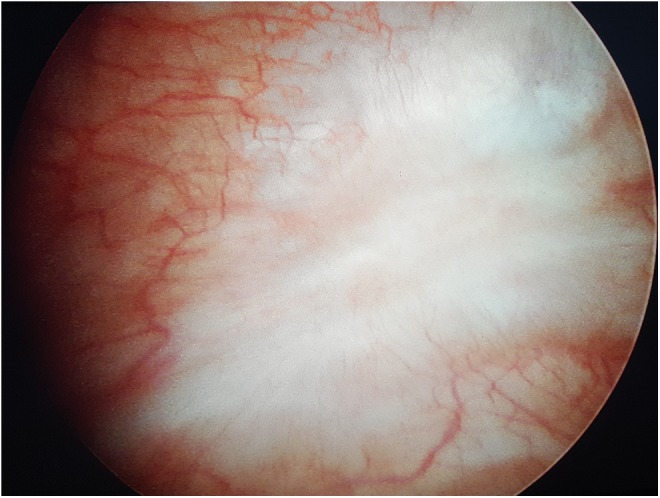
Postoperative cystoscopic appearance of the sealed vesicovaginal fistula.

The operative time was 65 minutes. Blood loss was minimal and could not be measured in the case. There were no intraoperative or postoperative complications. The patient was allowed oral intake within 3 hours, to move within 12 hours postoperatively, and was discharged after removal of the cystostomy on the first postoperative day. The Foley catheter was kept in place for 3 weeks. An oral anticholinergic was given until the removal of the Foley catheter. The patient was instructed to return to our office 3 weeks after surgery for urethral Foley catheter removal and subsequent cystoscopic and vaginal inspection to confirm VVF repair (Fig. 4). During a 3-month follow-up, the patient remained continent, and the laboratory results were within normal range.

## Discussion

There is currently no consensus on the surgical approach to VVF repair. At present, laparoscopic procedures tend to replace open surgery with comparable results. Laparoscopic VVF repair is beneficial over open surgery as the patient has less postoperative pain, lesser analgesic requirements, shorter recovery time, and shorter hospital stay.^1^ Although laparoscopic repair has excellent success, the major disadvantages associated with this technique are the difficulty in intracorporeal suturing, prolonged operation time, and steep learning curve. For small VVF, endoscopic conservative treatments have become increasingly popular, reducing the invasiveness of treatment and shortening the period of convalescence. Several minimally invasive techniques such as fulguration and curettage have been performed for repair of VVF. Endoscopic treatment of VVFs by fulguration of the fistulous tract is the most common minimally invasive method for small fistulas on day care basis. O'Conor applied electrocoagulation for highly situated small fistula with dimensions of 3.5 mm or smaller.^2^ Curettage of the fistula tract with screw followed by prolonged catheterization has been reported to be effective in a small series of patients by Aycinena.^2^ However, there is no publication about sealing of VVF tract in the literature.

Advanced bipolar energy devices such as LigaSure may be used to seal on arteries, veins, lymphatic vessels, and tissue bundles in a number of surgical specialties such as urology, gynecology, colorectal, and cardiothoracic fields. These are particularly advantageous for sealing vessels up to 7-mm in diameter through uniform compression and efficient energy delivery. In addition, they have greatly reduced the need for laparoscopic suture ligation, which is technically demanding and time consuming.^3^ These systems provide precise energy delivery and electrode pressure to tissues for a controlled time period to achieve a complete and permanent fusion of tissues and vessel lumens.

In most cases, laparoscopic procedures take a relatively long time. The mean operative time in the literature ranges from 110 to 330 minutes even in cases of robot-assisted laparoscopic repair cases.^4^ The operative time in our case was 65 min. Intraoperative difficulties were noted in the first case, which included grasping the fistula, pressure of insufflation to maintain the pneumovesicum during port insertion.

In general, there is no massive hemorrhage in VVF repair operations. There was no notable blood loss in our case either. Increased number of ports and internal dissections are the main cause of postoperative pain after laparoscopic repair. Four ports were used in many studies about repair of VVF, whereas more ports were used in others. In our case, VVF repair was performed using two ports only.

No matter which approach is performed, the surgeons believe that the most important issue of VVF repair remains a “water-tight seal,” and adequate bladder drainage after surgery to allow for tissue healing, as suggested by the literature. The authors think that there was a water-tight seal in the TBSF procedure.

## Conclusions

In select cases, TBSF may be effectively used for closure of small fistula tracts with a flatter learning curve, shorter operative time, less blood loss, decreased morbidity, improved cosmesis, and briefer hospital stay.

## Abbreviations Used

EBVS: electrothermal bipolar vessel sealing system
TBSF: transvesicoscopic bipolar sealing of the vesicovaginal fistula
VVF: vesicovaginal fistula
